# Engineering three-dimensional topological insulators in Rashba-type spin-orbit coupled heterostructures

**DOI:** 10.1038/ncomms2972

**Published:** 2013-06-06

**Authors:** Tanmoy Das, A. V. Balatsky

**Affiliations:** 1 Theoretical Division, Los Alamos National Laboratory, Los Alamos, New Mexico 87545, USA; 2 Center for Nanotechnologies, Los Alamos National Laboratory, Los Alamos, New Mexico 87545, USA; 3 Nordita, KTH Royal Institute of Technology and Stockholm University, Roslagstullsbacken 23, SE-106 91 Stockholm Sweden

## Abstract

Topological insulators represent a new class of quantum phase defined by invariant symmetries and spin-orbit coupling that guarantees metallic Dirac excitations at its surface. The discoveries of these states have sparked the hope of realizing non-trivial excitations and novel effects such as a magnetoelectric effect and topological Majorana excitations. Here we develop a theoretical formalism to show that a three-dimensional topological insulator can be designed artificially via stacking bilayers of two-dimensional Fermi gases with opposite Rashba-type spin-orbit coupling on adjacent layers, and with interlayer quantum tunneling. We demonstrate that in the stack of bilayers grown along a (001)-direction, a non-trivial topological phase transition occurs above a critical number of Rashba bilayers. In the topological phase, we find the formation of a single spin-polarized Dirac cone at the 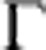-point. This approach offers an accessible way to design artificial topological insulators in a set up that takes full advantage of the atomic layer deposition approach. This design principle is tunable and also allows us to bypass limitations imposed by bulk crystal geometry.

## Abstract (web summary)

Presently, the design of 3D topological insulators is limited to single-compound synthesis with appropriate symmetries. Here, the authors propose a new design principle for 3D topological insulators based on stacked 2D Fermi gases, which may allow for better control of topological properties.

## Graphical abstract

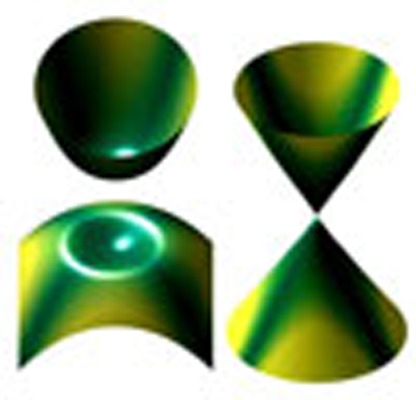

The unusual properties of topological insulators (TI) rely on synthesizing suitable materials that inherit various invariant symmetries and spin-orbit coupling in the bulk ground state, and that allow the formation of metallic Dirac fermions at the boundary^1^,^2^,^3^,^4^,^5^,^6^. From the theoretical standpoint, the bulk symmetries include a combination of low-lying odd parity orbitals, and time-reversal symmetry with a crystal geometry that can conspire an inverted band structure via strong spin-orbit coupling^7^,^8^, or crystal mirror symmetry^3^. The main focus of the search for TIs so far has been limited to the synthesis of bulk materials with these inherent characteristics. Compounds of choice are those with a large bulk spin-orbit insulating gap (Bi-based compounds^9^,^10^ and their functional variants^3^,^11^,^12^,^13^). The efforts have then been extended to manipulating the non-trivial topological phase by driving a trivial topological system through a topological phase transition via chemical doping^14^,^15^, by tuning the lattice constant^13^,^16^, or by introducing broken symmetry quantum phases^17^,^18^. These materials offer tremendous opportunities for applications as well as realizations of numerous non-trivial properties such as anti-localization, unusual magneto-electric effects and controlled electronic mass by an applied magnetic field or a proximity to quantum orders^1^,^2^,^19^,^20^,^21^,^22^. The presence of a Dirac cone at the Fermi level without the intervention of any bulk state will also make the TI an attractive candidate to realize the fractional quantum statistics and non-Abelions^1^,^2^,^7^,^21^,^22^. Still with all the exciting developments, it has proven to be challenging to obtain a true bulk insulator, or having a surface Dirac point at the Fermi level.

We propose here an alternative approach to design TIs by combining a set of layers of two-dimensional Fermi gases (2DFGs) with Rashba-type spin-orbit coupling. This two-dimensional spin-orbit locked metallic state, in the presence of interlayer quantum tunneling, translates into a bulk insulator with *Z*_2_-invariant topological properties and Dirac excitations on the surface. The idea is to grow two counter-helical Rashba-planes (dubbed ‘Rashba-bilayer’)—which hitherto imposes time-reversal invariance—along the (001)-axis with an interlayer distance that enables single-electron hopping between them. With an effective Hamiltonian, we observe that above a critical number of Rashba bilayers, ~5–6 layers for a realistic parameter choice, the non-trivial TI phase emerges. The resulting gapless single-Dirac cone has a linear slope determined by the Rashba-coupling strength and is thus externally tunable. We find that this layer-by-layer approach of Rashba bilayers has all the known properties of the bulk TI; for example, we present a direct calculation of the Chern index in the bulk to support the topological nature of the resultant state. A design principle for such Rashba bilayer with the help of ferroelectric substrates to the 2DFG is also proposed below. With the rapid expansion of surface growth techniques like molecular beam epitaxy, one would be able to explore a large set of compounds that might not be accessible in the bulk phase and yet possess TI behaviors.

In what follows, we lay a general framework for generating and manipulating a ‘homemade’ three-dimensional TI. In principle, one can envision to use the approach we propose and take it a few steps further. For example, a heterostructure setup allows one to introduce layers of magnetism^21^,^22^, superconductivity^19^,^20^,^23^ and other exotic many body orders^24^ within the topological matrix. Taken together, the search for TI materials that obey several symmetry properties and inherits spin-orbit coupling can thus be replaced with ‘homemade’ systems by generating spin-orbit coupling via external or internal electric fields, and by imposing symmetry properties via manipulating the heterostructure geometry. Such TI will be free from any particular crystal geometry studied earlier.^25^

## Results

### Rashba-bilayer heterostructure

**Figure 1:**
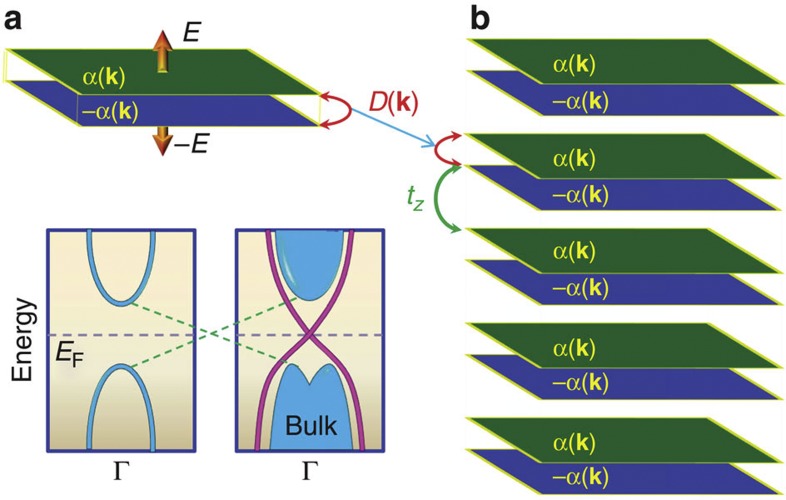
Rashba bilayer of 2DFGs and its heterostructure setup. (**a**) A bilayer combination of opposite Rashba-type spin-orbit coupling 2DFGs, denoted by *α*(**k**), −*α*(**k**), representing 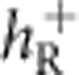 and 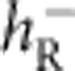, respectively in the Hamiltonian in Equation 1. *D*(**k**) gives the interlayer electron tunneling between them. (**b**) As grown Rashba bilayers with finite inter-bilayer coupling, *t*_*z*_. (**c**, **d**) Illustration of band dispersions for a bilayer 2DFG, and its heterostructure version, respectively. The emergence of an inverted band curvature in the valence bulk band marks the topological phase transition, as also demonstrated in first-principle bandstructure calculations^26^, and ARPES data^14^.

We start with depicting our basic idea in Fig. 1. The main idea relies on gluing two Rashba-type spin-orbit coupled 2DFGs with opposite signs of Rashba coupling, denoted by ±*α*(**k**). We take them to be close to each other such that quantum tunneling [*D*(**k**)] couples them, as illustrated in Fig. 1a. Such opposite-coupled Rashba-bilayer can easily be manufactured by creating a potential gradient between two 2DFGs with the help of gating, or by inserting oppositely polarized ferroelectric substrate between them, among others. As opposed to a metallic single-Rashba 2DFG, the Rashba bilayer opens an insulating gap, determined by the value of *D*(**k**), with a minimum gap at the 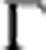-point. Then this Rashba-bilayer setup is to be repeatedly grown along the (001)-direction with an inter-bilayer electron hopping, *t*_*z*_, which is required to be different from *D*(0) to eliminate the degeneracy in the band structure. Above a critical number of the Rashba bilayers in such a heterostructure setup, a bulk topological phase transition commences. A good indicator of the non-trivial topology is the development of an inverted band dispersion or ‘dent’ in the valence Fermi sea, as illustrated in Fig. 1d. Such an inverted band dispersion is well established in first-principle band structure calculations^26^, and angle-resolved photoemission spectroscopy (ARPES) measurements^14^. Both analytically and numerically, we investigate a realistic parameter space and find that the state is a non-trivial topological state with a single Dirac cone, carrying all salient topological properties that were derived and realized earlier in bulk three-dimensional systems^1^,^2^. A detailed progression of the resulting band structure is given in the Supplementary Methods.

### Effective model for the Rashba-bilayer heterostructure

Based on the above-proposed setup, we now derive an effective low-energy theory. In each 2DFG planes, electrons with momentum **k** experience an effective anisotropic magnetic field, induced by an electric field *E*_*z*_, which couples to their spin *σ* to give rise to Rashba-type spin-split electronic bands 
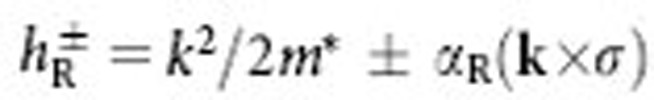
. Here *α*_R_ is the Rashba-coupling strength, controlled by the external or internal electric field, and m^*^ is the effective mass of electrons. **k** is defined in the 2D plane, and correspondingly, *σ* stands for Pauli matrices in the spin-subspace. We assume two such counter-propagating helical 2DFGs, 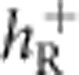 and 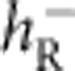, are grown close to each other. Because of wavefunction overlap, finite quantum tunneling *D*(**k**) between two 2DFGs is active. The next step in this setup is to grow these Rashba bilayers along *z* axis with a spin-conserving electron hopping between them, characterized by *t*_*z*_. Therefore, the general form of the effective Hamiltonian for *N* semi-infinite layers within an open-boundary condition can be expressed as


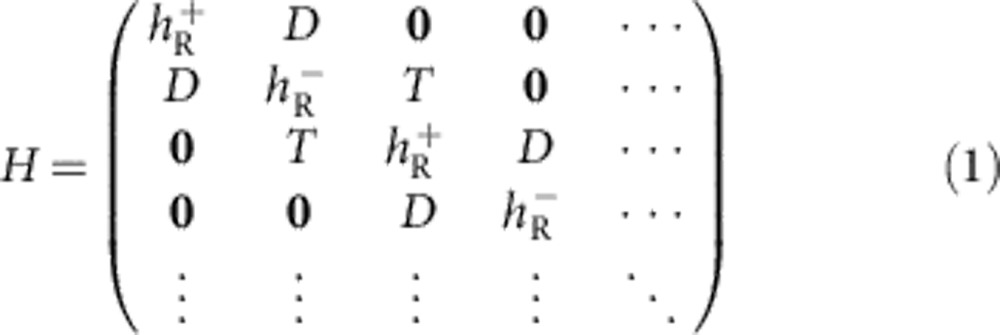


Each term in the above Hamiltonian is a 2 × 2 matrix. To keep the formalism general and readily tunable, we allow for anisotropic hopping between 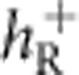 and 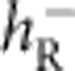 as 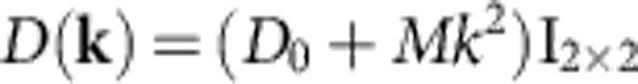, which can also be thought of as Dirac mass and Newtonian mass terms, respectively, due to their impacts on the band structure obtained. The tunneling between two adjacent bilayers is *T*=*t*_*z*_I_2 × 2_. **0** is a 2 × 2 zero matrix, and I_2 × 2_ is the identity matrix. Time-reversal invariance of the above Hamiltonian consequently emerges due to the fact that 
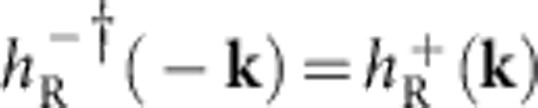
, which is an essential criterion for the formation of helical edge states with a Dirac point, endowing the system to the *Z*_2_ classification^4^,^5^,^6^,^7^,^8^,^27^.

The values of parameters m^*^ and *α*_R_ depend on the type of Rashba-setup under consideration, whereas those for the expansion parameters *D*, *M* and *t*_*z*_ are controllable mainly by the details of the heterostructure. An elaborated band structure progression as a function of these parameters is given in Supplementary Fig. S2. Here, we discuss the emergence of a topological bulk insulator with helical surface state for a representative value of m^*^=0.1 eV^−1^Å^−2^, and *α*_R_=1.5 eVÅ. Such a value of the Rashba parameter is readily achievable in Bi-based surface and quantum-well states, as well as in BiTeI bulk systems^28^. The Dirac mass has the same meaning as used for 2D quantum spin Hall (QSH) insulator^29^, and 3D TIs^8^, in that it is responsible for opening a band gap between the two adjacent time-reversal terms 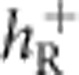 and 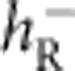 at the 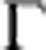-point inside the bulk. When several slabs of Rashba bilayers are glued together with *t*_*z*_ tunneling, the insulating gap persists in every layer, except on the boundary. Two helical edge states develop at each boundary and meet at a gapless Dirac cone above a critical value of the number of layers, *N*_c_.

**Figure 2:**
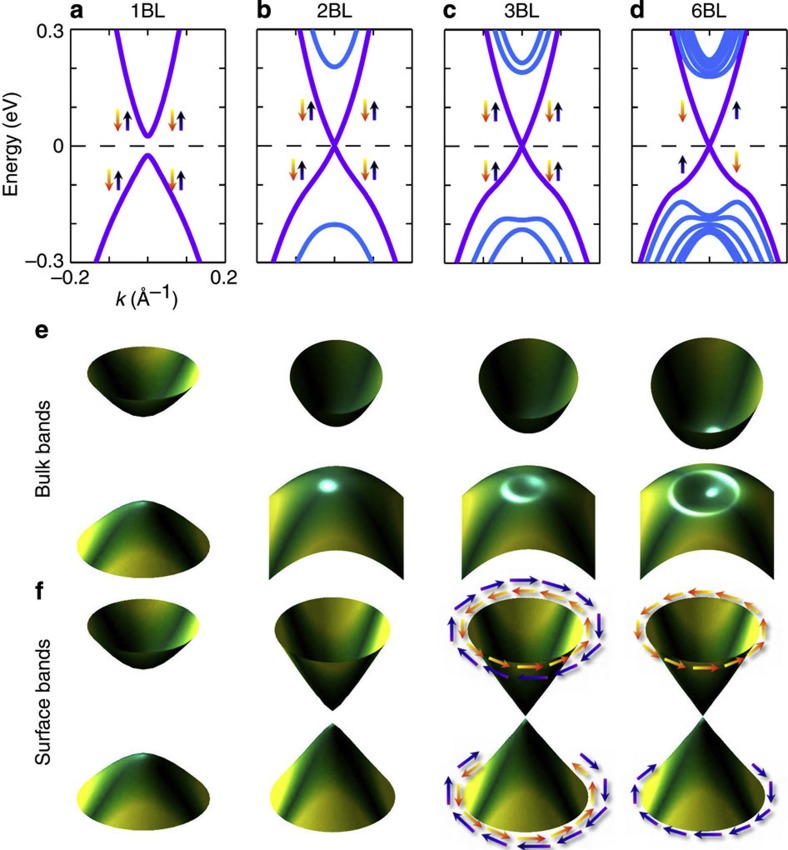
Band progression and formation of gapless surface states. (**a**–**d**) Evolution of band dispersion for a single (1BL) to six (6BL) of Rashba bilayers. (**e**–**f**) Corresponding 3D view of the band dispersions in the *k*_*x*_, *k*_*y*_-plane for the bulk (top panel) and surface (bottom panel) bands, respectively. The parameter set for this calculation is given in the text. In going from two to three bilayers, the bulk valence band topology reveals the emergence of an inverted shape (see also **c** and corresponding **f**), which indicates the topological phase transition from a trivial to the non-trivial phase. However, it takes about six bilayers to turn off the inter-edge tunneling to commence a gapless Dirac cone at the surface. Arrow dictates the spin orientation. The definite spin-chirality of the gapless Dirac cone is illustrated by counter-rotating arrows in **f**. Green (min) to yellow (max) colormap gives projected spin-orbit locking eigenstates.

The value of *N*_c_ depends only on the Dirac mass *D*_0_ and on the interlayer tunneling *t*_*z*_, but not on other terms, and thus is controllable solely by the layer-by-layer deposition technique. We illustrate this case for a representative value of *D*_0_=−25 meV, *t*_*z*_=−0.2 eV in Fig. 2. Above three or more Rashba bilayers, we observe, both numerically and analytically (see Supplementary Methods), that an inverted bulk insulating gap develops in the interior Rashba bilayers. This reflects in the transformation of the quantum-well-like states of the interior slabs into an inverted dispersion resembling a dent shape in the filled Fermi sea. We recall that this dent-like inverted valence band is a critical signature of the non-trivial topological phase, and has been consistently obtained in first-principle calculations^26^, and has also been observed in 3D TI materials^14^.

To further quantify the strong topological phase transition as a function of the number of layers, we compute the topological invariants *v* (or an axion angle parameter *θ*) of the Hamiltonian in Equation (1). As the Hamiltonian is invariant under inversion symmetry, we can derive the essential parity operator for each pair of Kramers degenerate valence bands from the constraint *PH*(**k**)*P*^−1^=*H*(−**k**), with 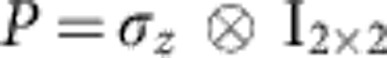, for each Rashba bilayer. Thus, we evaluate the topological quantum index as^30^


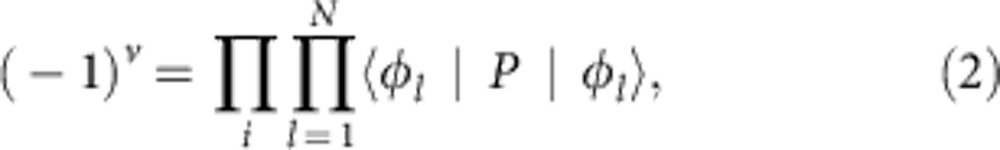


where *i* stands for the time-reversal high-symmetry points on the Brillouin zone, and 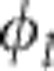 are the eigenvectors for each layer *l*. For the parameter set used, we indeed find that a parity inversion occurs at *N*_c_, which, according to the *Z*_2_ topological criterion^4^,^7^,^8^,^27^, marks the emergence of the topological insulating phase at this critical value of *N*. Above this critical thickness, the parity value stabilizes to the non-trivial TI^31^.

A consequence of the *Z*_2_ TI is the presence of a spin-polarized Dirac cone at the surface. Despite the emergence of a non-trivial topological phase above three Rashba bilayers, a surface gap persists due to finite quantum tunneling between the two edges, and the massless Dirac point appears above six layers for this parameter choice. Such a formation of massive Dirac quasiparticles below a critical value of quintuple layers is observed in Bi_2_Se_3_ thin films^23^, and also in doped bulk TlBi(S_1−*x*_Se_*x*_)_2_^14^,^15^.

**Figure 3:**
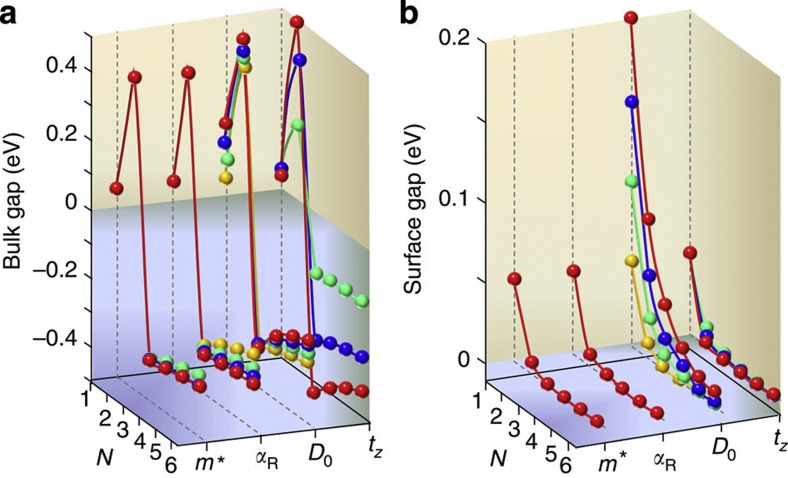
Bulk and surface gaps in a realistic parameter space. (**a**) The critical number of layers, *N*_c_, at which an inverted bulk gap opens. The bulk band gap jumps from *D*(0) at *N*=1 (equivalent to *t*_*Z*_=0) to a large positive value, and then consistently becomes negative for N≥3, constrained by the Hamiltonian (see also Supplementary Fig. S2). For each parameter set, the corresponding other parameters are set to be constant to the values mentioned in the main text. The varying parameters are: m^*^=1 (green), 2.5 (blue), 5 (red) in eV^−1^Å^−2^. *α*_R_=1 (orange), 1.5 (green), 2 (blue), and 3 (red) in eVÅ that are attainable in existing systems.^28^ *D*_0_=−25 (orange), −50 (green), −75 (blue), and −100 (red) in meV, and *t*_*z*_=−100 (green), −200 (blue) and −500 (red) meV. As *Mk*^2^=0 at the 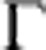-point, the parameter *M* does not have any effect on the direct gap structure. (**b**) Corresponding surface gaps at the 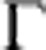-point. Clearly, the gapless surface Dirac cone formation depends on two parameters, the interlayer hybridization *t*_*z*_, and strongly on the Dirac mass *D*. The parameter values here are same as in **a**.

We tabulate the values of bulk and surface gaps as a function of various tuning parameters in Fig. 3. As expected, the electron’s mass m^*^ and Rashba-coupling *α*_R_ do not have any significant effect on the gap values, and thus give us an alternative approach for generating bulk TIs beyond atomistic spin-orbit coupling and heavy electron mass. The Dirac mass *D* and interlayer tunneling provide the tuning knob for engineering the surface and bulk gaps, which are readily tunable via heterostructure details. In Supplementary Fig. S2, we show the corresponding energy dispersions for a large range of realistic parameters.

### Surface Dirac cone properties

We can formulate the Hamiltonian for the edge state by using the theory of invariants. The time-reversal symmetry imposes that a spin-up state at momentum **k** must be entangled to a spin-down state at −**k** via spin-orbit coupling. As a consequence of this, according to the Fermion doubling theorem, a gapless Dirac point is guaranteed at the 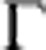-point. In what follows, two counter-propagating surface states form with one spin state from the upper Rashba-term (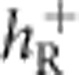), and an opposite spin state from the lower Rashba term (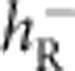), which are related by time-reversal operation. By projecting the full Hamiltonian in Equation (1) onto a single slab, we can write down the surface Hamiltonian in this basis to leading order in *k* as


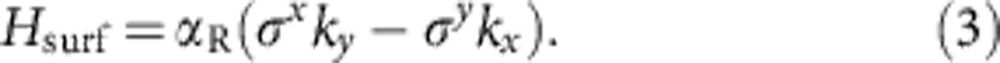


Note that the similar-surface dispersion is also obtained for 3D TIs^7^,^8^, except that here its slope is solely determined by the Rashba-coupling strength. Therefore, the velocity of the Dirac fermions turns out to be 
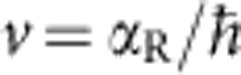
, and externally tunable. For values of the Rashba-coupling constant as large as 3.8 eV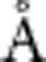, achieved to date in bulk BiTeI (ref. 28), we get 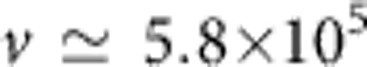 m s^−1^, which is several order of magnitude larger than the value achieved so far in 3D TIs^9^,^32^. The energy scales above which the slope of the surface state deviates from a linear-in-energy to a power-law behavior depends on the value of mass term *D*(0). For the parameter set used above, we obtain a linear dispersion expanding about 0.3 eV on both sides of the Dirac point, which is also tunable (see Supplementary Fig. S2).

The spin-polarized surface states can be imaged directly by ARPES and tunneling spectroscopies, as well as via transport measurements. An interesting transport property of Dirac fermions is the quantum Hall effect that can be achieved by inserting a magnetic layer in the heterostructure next to the boundary, so that it breaks time-reversal symmetry on the surface via the proximity effect, but not in the bulk^7^,^21^. We can express the surface and magnetic layer Hamiltonian in a combined d-vector form as **d**·**σ**, where the time-reversal invariant components are *d*_*x*_=−*α*_R_*k*_*x*_ and *d*_*y*_=*α*_R_*k*_*y*_, and time-reversal breaking *d*_*z*_ gives the Zeeman energy splitting due to induced magnetization. Without the presence of a *d*_*z*_ term, the winding number of the upper and lower chiral states, defined as 
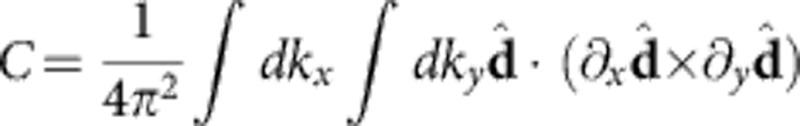
 (the notation ‘hat’ represents corresponding unit vector), where the integration is performed over a closed loop around the Dirac cone, cancel each other. However, when a broken time-reversal symmetry is imposed, *C* becomes equal to ±*σ*_*z*_, where *σ*_*z*_ is the component of the spin along the magnetic field orientation. In this case, quantized spin-Hall conductance becomes fractional in units of *e*^2^/*h* as 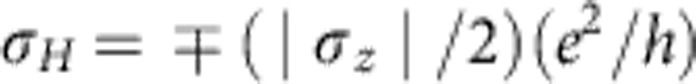. For a fully polarized spin-configuration along the magnetic field, the above equation generates a half-integer anomalous quantum-Hall effect (QHE). The fractional or half-integer QHE is a trademark feature of Dirac systems^1^,^2^,^7^,^32^,^33^, and can be used as a test of our proposal. An interesting consequence of the half-integer Landau level is that as the time-reversal breaking mass term, *d*_*z*_, approaches zero, the counter-propagating Landau levels move to zero energy, and the system can act as protected quantum Hall insulator despite the presence of energy states at the Fermi level^34^. This unique scenario has been discussed theoretically for TIs, but never been realized experimentally due to the absence of an isolated Dirac point at *E*_F_ (ref. 32); however, it can be realized in the present heterostructure setup.

### Design principles for Rashba bilayers

**Figure 4:**
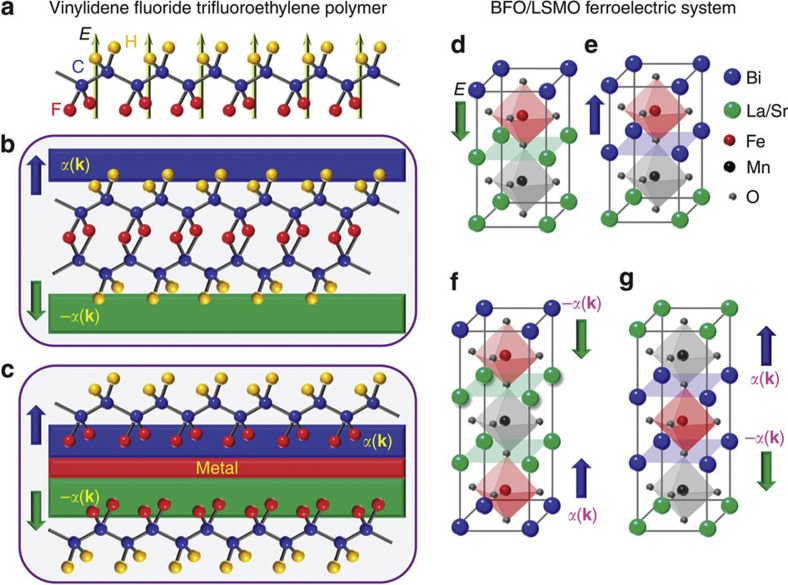
Design principles for counter helical Rashba bilayer via ferroelectric substrates. (**a**) In the left panel, we show the structure of a copolymer of vinylidene fluoride with trifluoroethylene, P(VDF-TrFE) which exhibits ferroelectricity even in its two-dimensional films^36^. Two oppositely oriented such films can be used to generate oppositely polarized Rashba spin-orbit couplings in the adjacent layers of 2DFGs. Two possible design principles using this polymer are presented in **b** and **c**. Blue and green arrows depict opposite directions of electric field. ±*α*(**k**) distinguish two 2DFGs with opposite Rashba-type spin-orbit coupling. (**d**–**g**) In the right panel, we demonstrate the design principles of Rashba bilayer by exploiting the ferroelectric BFO/LSMO superlattice^39^. Here, the existing Bi-layers are expected to possess an oppositely polarized Rashba spin-orbit coupling via manipulating the direction of the inversion symmetry breaking inside them.

Finally, we propose two representative design principles for the counter-polarized Rashba-type spin-orbit coupling bilayers in Fig. 4. Of course, the materials fabrication of them is not limited to these two design methods, and one can envision different methods by exploiting our general idea of invoking a polarized medium inside the Rashba-bilayer. Here, we suggest to use the oppositely polarized ferroelectric materials as the substrate to two adjacent 2DFGs. The idea is to utilize the uniform polarization of a ferroelectric material to tune the Rashba-type spin-orbit coupling at the interface^35^. We think of systems such as ferroelectric polymers and BiFeO_3_/La_1−*x*_Sr_*x*_MnO_3_ (BFO/LSMO) superlattice as possible candidates for such substrates that come with an additional benefit that they are highly strain-free. Another crucial advantage of this design principle is that the ferroelectricity is intrinsic in these these materials, and it is generated by the inversion symmetry breaking in the bulk. Thus one can easily generate oppositely aligned ferroelectric polarization on both edges by creating a reverse inversion symmetry breaking with respect to a common mirror *ab*-plane between them.

In our first materials specific set up, we propose to use a copolymer of vinylidene fluoride with trifluoroethylene, P(VDF-TrFE), consisting of -((-CF_2_-CH_2_)_*x*_-(-CF_2_-CHF-)_1−*x*_)_*n*_- chains, controlled by the C–C bonds between fluorine pairs^36^, as shown in Fig. 4a. This polymer exhibits spontaneous polarization as large as 0.1 cm^−2^, and a hysteresis loop for switching polarization even in two monolayer films^37^. The polarization is highly homogeneous and tunable. The electric field is generated from fluorine to hydrogen ions due to the lack of inversion symmetry in the polymer as illustrated in Fig. 4a. This electric field can be exploited in two ways to generate a Rashba bilayer. In Fig. 4b, we show that two P(VDF-TrFE) polymers can be attached back to back (setting either fluorine or hydrogen layer as a mirror plane). If a 2DFG with heavy elements such as Bi is attached on both sides of this polymer, they may generate opposite Rashba-type spin-orbit couplings. In this case, as the mirror plane intrinsically maintains a constant charge character, two opposite electric fields are unlikely to annihilate each other. On the other hand, as the dc conductivity is quite large in this film^38^, a finite electron tunneling between the two Rashba-layers can turn on due to the wavefunction overlap. This setup will then work as Rashba bilayers of interest.

In Fig. 4c, we propose a second Rashba bilayer design scenario using this polymer. Here, two polymers can be attached from the top and the bottom sides of the 2DFG-bilayers, enabling an opposite Rashba spin-orbit coupling. In between them, one needs to add a metallic substrate that has a polar surface (having opposite polarity on both sides may make a metal even more suitable here). As this polymer is very flexible, we can expect to obtain a strain-free, homogeneous counter helical Rashba-type spin-orbit coupling on two adjacent 2DFGs.

In the second setup, we propose to use a ferroelectric BFO/LSMO superlattice. Here, the interfacial valence mismatch between BFO and LSMO (without any significant structural mismatch) influences the electrostatic potential step across the interface, which manifests itself as the bias-voltage in the ferroelectric hysteresis loops. As shown in Fig. 4d and e, in this material, the polarization is reversible depending on how LSMO layer is attached to the BFO layer^39^. We can exploit these properties to generate two Bi-layers with opposite polarizations, as demonstrated in Fig. 4f and g. The added benefit here is the strong spin-orbit coupling of Bi atoms, which will thus provide an opposite Rashba-type spin-orbit coupling in two adjacent Bi-layers. As LSMO can be doped easily from paramagnetic to semimetal to (trivial topological) insulator phase, the interlayer hopping is easily tunable in this setup. As the BFO/LSMO interface heterostructures are readily grown, our proposed BFO/LSMO/BFO structure can also be expected to be achievable in the same way. Despite the fact that the magnetic moment in BFO is mainly on the Fe sites, it may be suggestive to avoid the magnetic ground state of BFO to achieve a full spin-polarization of the Rashba-type spin-orbit effect. The possibility of acquiring a ferroelectricity controlled Rashba spin-orbit coupling in BaTiO_3_ as demonstrated by first-principle calculation^35^ also makes it a suitable candidate for generating a topological insulator in the superlattice structure with LSMO. Finally, we note that the infinitely adaptive superlattice phase of single layers of Bi and Bi_2_Se_3_, as opposed to bilayers of each of them used in the study by Valla *et al.*^40^, which is a topological semimetal, can become a topological insulator.

## Discussion

Our proposal clearly overcomes the limitation of searching for suitable combinations of crystal geometry, and inherent wave function symmetries within a bulk system to obtain TI. The present formalism is free from any particular crystal geometry, and thus provides a widespread playground for engineering ‘homemade’ TIs with surface Dirac state properties. Another advantage of the present case is that here the edge state is isolated from the bulk states, instead of being buried inside the bulk Fermi sea, which has so far significantly limited the usage of existing 3D TIs. The heterostructure, with depositing capability of one atomic layer at a time, can also easily accommodate magnetic and superconducting layers, beyond the conventional doping or proximity effects. It offers a freedom of bringing the desired components to the relevant part of a nanostructure by demand. This ability thus will promote a unambiguous detection of non-Abelian particles^19^,^21^,^22^ and anomalous Hall effect. Given that the electron interaction is strong and tunable in 2DFG (ref. 24), novel broken symmetry phases are easy to yield in this heterostructure setup than in a weakly correlated 3D TI.

## Author contributions

The present research stemmed from fruitful discussions between the authors. Both authors contributed to the writing of the manuscript.

## Additional information

**How to cite this article:** Das, T. & Balatsky, A. V. Engineering three-dimensional topological insulators in Rashba-type spin-orbit coupled heterostructures. *Nat. Commun.* 4:1972 doi: 10.1038/ncomms2972 (2013).

## Supplementary Material

Supplementary Information Supplementary Figures S1 and S2, Supplementary Methods and Supplementary References
