# Supplementary material for: Engineering three-dimensional topological insulators in Rashba-type spin-orbit coupled heterostructures
Source: Nat Commun. 2013 Jun 6;4:1972. doi: 10.1038/ncomms2972 (PMC3709477; doi:10.1038/ncomms2972)
Supplement: Supplementary Information — Supplementary Figures S1 and S2, Supplementary Methods and Supplementary References [file ncomms2972-s1.pdf]

Supplementary Information for "Engineering three-dimensional topological insulators in Rashba-type spin-orbit coupled heterostructures"

Tanmoy Das, A. V. Balatsky

Suplementary Figures

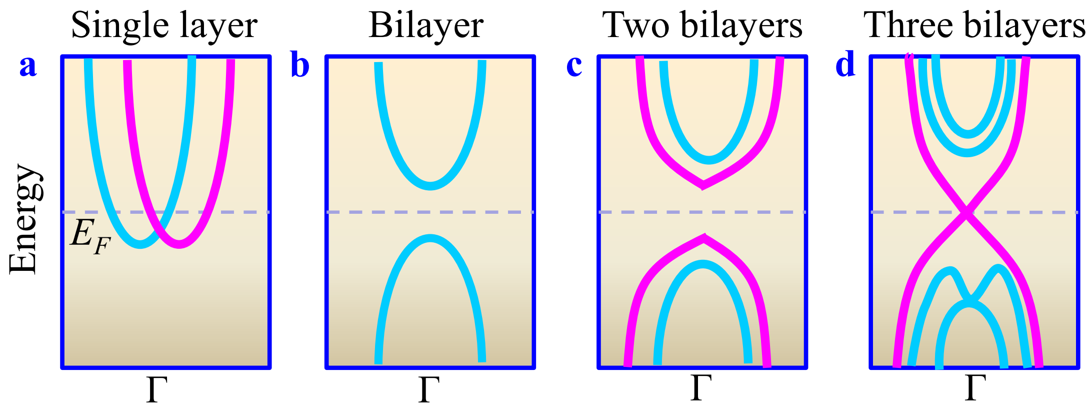

Supplementary Figure S1: Band progression from a single to a bilayer to a heterostructure of Rashba-type spin-orbit coupling 2DFGs. Schematic drawings of the band curvatures expected for a single- to a bi- to a multiple layers of 2DFGs.

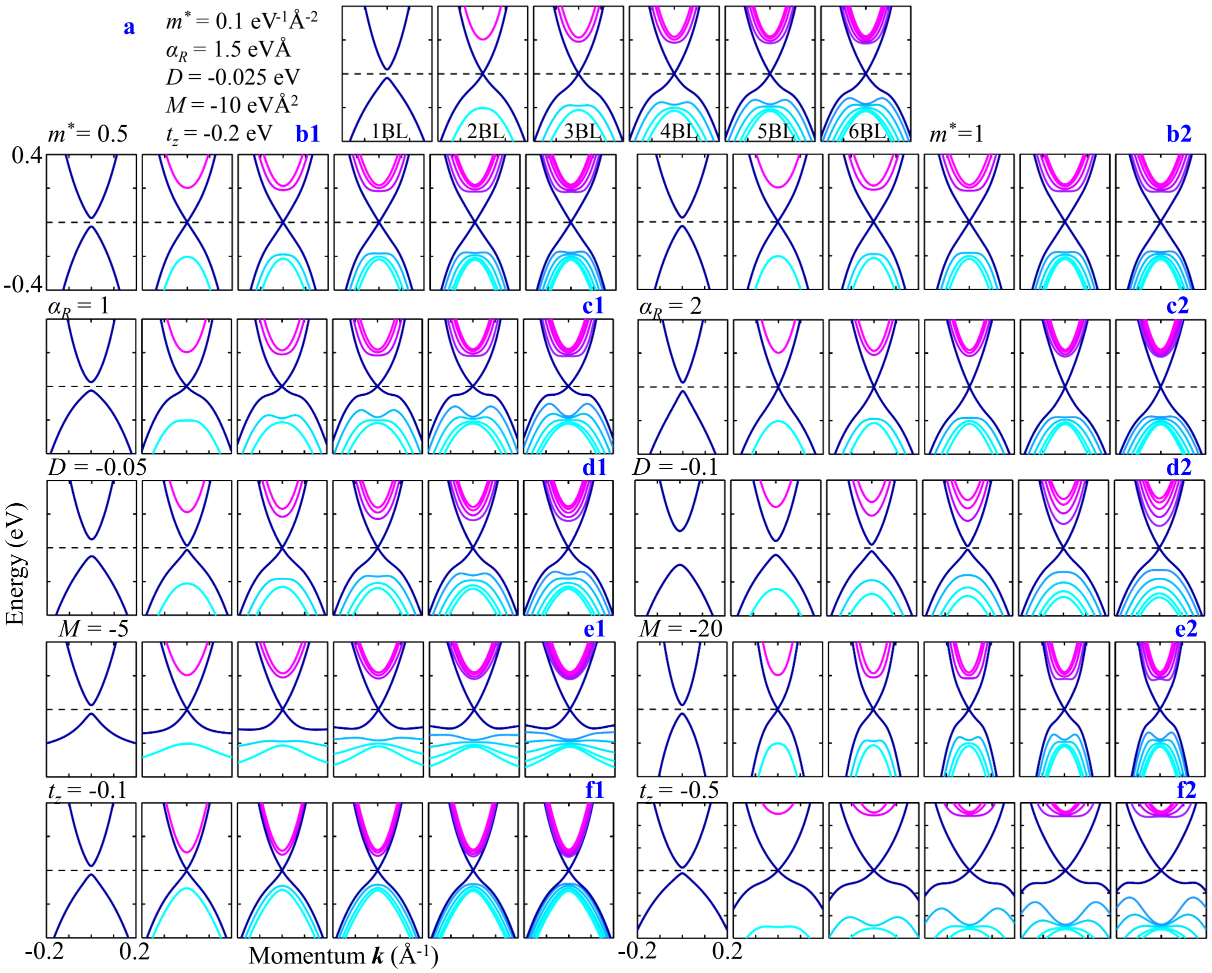

Supplementary Figure S2: Variations of the dispersion relation for a wide range of parameter space. a, The band dispersion for a bilayer 2DFG to six layers setup as shown in Fig. 1 of the main text. In the rest of the figures, only a single parameter is tuned (in each row), while keeping others parameters same to that in **a**. In each figure, the horizontal axis spans within the k_x = -0.2 to 0.2 Å^{-1} range at $k_y = 0$ and the vertical axis runs from E =-0.4 to 0.4 eV with Fermi level (E_F) lying at the center (black dashed line), except in **f2**, where the energy scale runs from -0.6 to 0.6 eV. The value of N_c for the formation of gapless surface state depends only on t_z and D_0, whereas the slope of the surface state is controlled by the Rashba-type SOC α_R (compare **b**, **c1**, and **c2**), and the span of the linearly dispersing surface state depends on the bulk insulating gap, as well as on the Newtonian mass M, as expected form Eq. 2 of the surface Hamiltonian.

Suplementary Methods

• Band dispersion for a Rashba bilayer

We provide further insights into the emergence of the TI from an analytical description. The Rashba-type SOC splits a non-interacting band into two in-plane spin-polarized bands. To keep the formalism general and concrete, we take a 2DFG, with a quadratic band, $k^2/2m^*$, where m^* is the effective mass of electrons. Under Rashba-type SOC,[41] the split bands are obtained by solving $h_{\mathrm{R}}^{\pm} = k^2/2m^* I_{2\times 2} \pm \alpha_{\mathrm{R}}(k_y\sigma_x + k_x\sigma_y)$, where α_{R} is the Rashba-type SOC. For simplicity, we define the second term as $\alpha(\mathbf{k})$. Next we create a Rashba bilayer with two counter-propagating helical 2DFGs, h_{R}^{+} and h_{R}^{-} in which a finite quantum tunneling is turned on, as denoted by $D(\mathbf{k})$. The Hamiltonian for the Rashba bilayer can be written explicitly as

$$H = \begin{pmatrix} k^2/2m^* & \alpha(\mathbf{k}) & D(\mathbf{k}) & 0 \\ \alpha^{\dagger}(\mathbf{k}) & k^2/2m^* & 0 & D(\mathbf{k}) \\ D(\mathbf{k}) & 0 & k^2/2m^* & -\alpha(\mathbf{k}) \\ 0 & D(\mathbf{k}) & -\alpha^{\dagger}(\mathbf{k}) & k^2/2m^* \end{pmatrix}. \tag{S1}$$

The resulting eigenvalues are two-fold degenerate: $E^{\pm}(\mathbf{k}) = k^2/2m^* \pm \sqrt{\alpha^2(\mathbf{k}) + D^2(\mathbf{k})}$. This specific bilayer setup helps generate two upward and downward dispersing bands, with a direct gap between them defined by $D(\mathbf{k})$ at Γ-point (since $\alpha(0) = 0$), see Supplementary Fig. S1b. $D(\mathbf{k})$ shall be even under inversion, e.g. $D(\mathbf{k}) = D_0 + Mk^2$, which destroys the spin-polarization, implying that the gap between the band bands is charge insulating.

• Band dispersions for two Rashba-bilayers

As depicted in Fig. 1 of the main text, we now take two Rashba bilayers, grown along the (001)-direction such that a quantum tunneling, say t_z, between the adjacent layers becomes active. The explicit form of the Hamiltonian for this case is

$$H = \begin{pmatrix} k^2/2m^* & \alpha(\mathbf{k}) & D(\mathbf{k}) & 0 & 0 & 0 & 0 & 0 \\ \alpha^{\dagger}(\mathbf{k}) & k^2/2m^* & 0 & D(\mathbf{k}) & 0 & 0 & 0 & 0 \\ D(\mathbf{k}) & 0 & k^2/2m^* & -\alpha(\mathbf{k}) & t_z & 0 & 0 & 0 \\ 0 & D(\mathbf{k}) & -\alpha^{\dagger}(\mathbf{k}) & k^2/2m^* & 0 & t_z & 0 & 0 \\ 0 & 0 & t_z & 0 & k^2/2m^* & \alpha(\mathbf{k}) & D(\mathbf{k}) & 0 \\ 0 & 0 & 0 & t_z & \alpha^{\dagger}(\mathbf{k}) & k^2/2m^* & 0 & D(\mathbf{k}) \\ 0 & 0 & 0 & 0 & D(\mathbf{k}) & 0 & k^2/2m^* & -\alpha(\mathbf{k}) \\ 0 & 0 & 0 & 0 & 0 & D(\mathbf{k}) & -\alpha^{\dagger}(\mathbf{k}) & k^2/2m^* \end{pmatrix}. \tag{S2}$$

The eigenvalues of Supplementary Eq. (S2) are given by

$$E_{\pm}^{\pm}(\mathbf{k}) = \frac{k^2}{2m^*} \pm \frac{1}{2}\left[2t_z^2 + 4\left(\alpha^2(\mathbf{k}) + D^2(\mathbf{k})\right) \pm 2t_z\sqrt{t_z^2 + 4D^2(\mathbf{k})}\right]^{1/2}. \tag{S3}$$

The energy dispersions imply that whereas a large bulk gap opens between E_{+}^{+} and E_{-}^{-}, the gap between the lowest energy states E_{-}^{+} and E_{+}^{-} decreases from their single bilayer value of D_0. Although, mathematically, there exists a solution for the values of D_0 and t_z at which the surface gap can be closed, but

there is no clear evidence of the bulk band party inversion for this Hamiltonian, and thus the resulting solution remains to be a trivial topological phase. The absence of the band inversion is also evident in the parity calculation and the parabolic shape of the band dispersion as presented in the main text.

• Band dispersions for three Rashba-bilayers

With three or more bilayers, coupled by finite quantum tunneling, the bulk systems begin to form in which the interior bilayer(s) acts as a bulk lattice where the edge layers give rise to the surface states. We show here that a bulk band inversion occurs for three bilayers, and thus promotes a non-trivial topological insulating phase. However, to get rid of the electron hopping from the two edges, or in other words, to close the surface gap, one requires a higher number of bilayers.

The analytical solution gradually becomes complicated and long with further increase of bilayers. However we can gain some insights into how an inversion occurs in three bilayers of 2DFGs by the symmetry of the Hamiltonian, irrespective of parameter choices. The Hamiltonian can be easily generalized from the Supplementary Eq. (S2), and we present the eigenvalue for some of the relevant bulk bands as,

$$\begin{aligned} E^{\pm}(\mathbf{k}) &= \frac{k^2}{2m^*} \pm \frac{1}{3X(\mathbf{k})}\left[3\left(3\alpha^2(\mathbf{k}) + 2t_z^2 + 3D(\mathbf{k})\right)X^2(\mathbf{k}) + 6t_z^2\left(t_z^2 + 6D(\mathbf{k})\right)X(\mathbf{k}) \right. \\ & \left. \qquad +6\left(3Y(\mathbf{k}) - 2\right)t_z^6 + 54t_z^4 D^2(\mathbf{k})\right]^{1/2}, \end{aligned} \tag{S4}$$

where

$$\begin{aligned} X(\mathbf{k}) &= t_z\left[-8t_z^2 + 36t_z D(\mathbf{k}) + 12Y(\mathbf{k})\right]^{1/3}, \\ \text{and} \qquad Y(\mathbf{k}) &= D(\mathbf{k})\left[-96D^4(\mathbf{k}) - 39t_z^2 D^2(\mathbf{k}) - 12t_z^4\right]. \end{aligned} \tag{S5}$$

The important message of the Supplementary Eq. (S4) is that there is a prefactor $1/X \sim 1/t_z(...)$ which enables an inverted band gap opening for a negative value of t_z. The resulting parity inversion in the valence band endows a non-trivial topological phase transition at a large parameter space (see Fig. 3 of main text). A visual proof of the inverted band gap can be obtained from the associated inverted band curvature of 'dent' shape in the valence bulk band, as illustrated in Supplementary Fig. S1c. According to Fu-Kane criterion,[7] for such case gapless Dirac states appear at the surface. However, for most values of the parameters shown in Supplementary Fig. S2, a finite gap at the surface still persists due to the finite size-effect, and the surface gap closing requires a characteristic number of Rashba-bilayers.

• Dependence of bulk and surface band structure on various parameters

For the benefit of understanding the role of each parameter on the bulk and surface band structures, and also for the material growth purpose, we present results for a systematic tuning of various parameters as a function of number of layers in Supplementary Fig. S2. The same results present in Fig. 2 of the main text is reproduced here in Supplementary Fig. S2a and the parameters are listed in the left side of this panel. For each row in the figures below, a single parameter is tuned while the others are kept to the value listed in Supplementary Fig. S2a.

• m^*: The effective mass m^* of the electrons have any significant effect neither on the bulk and the

surface band structure, nor on the slope of the emergent surface state. Since this quadratic term goes to zero at the Γ-term, it begins to play a role at higher energy, and with increasing m^*, it only slightly reduces the indirect bulk gap.

• α_R: An added benefit of the present proposal is that the Rashba SOC does not significantly affect the bulk or the surface gaps (very minor contribution to the indirect bulk gap), in contrary to the 3D bulk materials in which the bulk insulator gap is mainly controlled by the SOC. However, as deduced in the main text in Eq. 3, $\alpha_{\rm R}$ determines the velocity of the Dirac fermions on the surface. Therefore, for even a smaller value of $\alpha_{\rm R}$, the 'non-trivial' topological insulator and the Dirac surface state occurs, only the velocity and the linear dispersion energy span deceases with $\alpha_{\rm R}$. As tabulated in Ref. 28, in 1 or 1/3 monolayer of Bi, which is measured to give a 2DFG (not a 'surface' state of a 3D bulk system), one can achieve $\alpha_{\rm R}$ to be as large as 2.5-3 eVÅ. Assuming that in our proposed Rashba bilayer, this strength gets reduced by 1/3 or 1/2, one can still achieve our parameter set.

An interesting case of inhomogeneous Rashba-spin-orbit coupling can be mentioned here. Local fluctuations of Rashba SOC can be both a blessing and a curse to our proposal. If the Rashba SOC is spatially modulated in such a way that both counter-helical Rashba states can be achieved within a single layer, this can replace the effort to create our proposed Rashba bilayers. In one-dimensional systems, the possibility of a negative/positive switching effect of spin polarization via tuning spatially modulated Rashba SOC is studied.[42]

On the other hands, if the local fluctuation of Rashba SOC is highly inhomogeneous, it can lead to a reduction of the spin-polarization and an uneven gap structure in each layer, which is a curse to the topological properties. However, if this gap opening can be reduced to an extent that topological properties are retained, inhomogeneous Rashba SOC can be useful. There are theoretical proposals that inhomogeneous Rashba SOC can induce non-trivial phenomena such as magnetoelectric effect, noncentrosymmetric superconductivity[43], which can be realized within the topological matrix.

• t_z: As we have shown in Fig. 3, the inter-bilayer overlap matrix element t_z mainly controls the bulk insulating gap. Even for t_z as small as 100 meV, we get an insulating gap of about 200 meV. It should be noted that the actual hopping energy can be improved in two ways. While t_z is the overlap matrix-element, the actual electronic hopping is $E_z = t_z \exp{(ik_z d)}$, where d is the interlayer distance. Therefore, by reducing d, one can enhance E_z up to a critical limit for a given setup, which will not kill the opposite Rashba couplings between them. The second and more important mechanism is to increase the effective gap for a given value of parameters by increasing the number of bilayers, N, in the heterostructure.

• D_0: Unlike t_z, D_0 does not have any significant effect on the bulk gap, but it controls the surface gap. However, as shown in Fig. 3, surface gap can easily be closed by increasing the number of bilayers, N. In our results, we have used the values of t_z and D_0 as in the range of 10-300meV. This small value of hopping is easily achievable even for the case of a weak Van-der Waals interaction between the two layers.

• M: The Newton mass M is associated with a quadratic momentum dependence, and thus does not participate in the gap opening, and it contributes at higher energy. Higher the value of M, better the band dispersion topology.

Finally, it should also be noted that the 'not-trivial' bulk insulator and surface Dirac fermion properties are the manifestation of the symmetry invariance of the Hamiltonian, not a numerical result for a set of parameters. The results presented are calculated using a realistic parameter range, which are indeed achievable in realistic materials.

Suplementary References

[41] Bychkov, Y. A. & Rashba, E. I. Properties of a 2D electron gas with lifted spectral degeneracy. *JETP Lett.* **39**, 78-81 (1984).

[42] Gong, S. J., & Yang, Z. Q. Spin filtering implemented through Rashba spin-orbit coupling and weak magnetic modulations. *J. App. Phys.* **102**, 033706 (2007).

[43] Aoyama, K. & Sigrist, M. Model for magnetic flux patterns induced by the influence of in-plane magnetic fields on spatially inhomogeneous superconducting interfaces of $LaAlO_3$-$SrTiO_3$ bilayers. *Phys. Rev. Lett.* **109**, 237007 (2012).
